# A Diffusive Gradient-in-Thin-Film Technique for Evaluation of the Bioavailability of Cd in Soil Contaminated with Cd and Pb

**DOI:** 10.3390/ijerph13060556

**Published:** 2016-06-02

**Authors:** Peifang Wang, Teng Wang, Yu Yao, Chao Wang, Cui Liu, Ye Yuan

**Affiliations:** Key Laboratory of Integrated Regulation and Resource Development on Shallow Lakes, Hohai University, Nanjing 210098, China; wangjkdhg@163.com (T.W.); cwang@hhu.edu.cn (C.W.); liucuiqd@sina.com (C.L.); yuanyesun@126.com (Y.Y.)

**Keywords:** diffusive gradients in thin films (DGT), chemical extraction methods, combined pollution, cadmium bioavailability, wheat and maize

## Abstract

Management of heavy metal contamination requires accurate information about the distribution of bioavailable fractions, and about exchange between the solid and solution phases. In this study, we employed diffusive gradients in thin-films (DGT) and traditional chemical extraction methods (soil solution, HOAc, EDTA, CaCl_2_, and NaOAc) to determine the Cd bioavailability in Cd-contaminated soil with the addition of Pb. Two typical terrestrial species (wheat, *Bainong AK58*; maize, *Zhengdan 958*) were selected as the accumulation plants. The results showed that the added Pb may enhance the efficiency of Cd phytoextraction which is indicated by the increasing concentration of Cd accumulating in the plant tissues. The DGT-measured Cd concentrations and all the selected traditional extractants measured Cd concentrations all increased with increasing concentration of the addition Pb which were similar to the change trends of the accumulated Cd concentrations in plant tissues. Moreover, the Pearson regression coefficients between the different indicators obtained Cd concentrations and plants uptake Cd concentrations were further indicated significant correlations (*p* < 0.01). However, the values of Pearson regression coefficients showed the merits of DGT, CaCl_2_, and *C*_sol_ over the other three methods. Consequently, the *in situ* measurement of DGT and the *ex situ* traditional methods could all reflect the inhibition effects between Cd and Pb. Due to the feature of dynamic measurements of DGT, it could be a robust tool to predict Cd bioavaiability in complex contaminated soil.

## 1. Introduction

Cadmium is a common impurity in fertilizers and can contribute to accumulation of Cd in the soil. Due to human activities such as mining, smeltering, industrial emissions, and the use of low-quality fertilizers, at least half of agricultural soils have Cd contamination [1]. Due to its high toxicity, Cd is recognized as a class-one carcinogen by the International Agency for Research on Cancer (IAPC) [2]. Cd can have serious human health impacts through the mechanisms of bioaccumulation and bioamplification. The list of top 20 hazardous substances of the United States Environmental Protection Agency (USEPA) and the Agency for Toxic Substances and Disease Registry (ATSDR) include Cd [3]. However, traditional measurements such as total concentration of target elements in soil do not reflect the potential for impacts resulting from ecotoxicity [4]. The bioavailable fractions of metals may be related to the concentration of these elements that occur as ions and to their kinetically labile species [5]. In the past years, the potential bioavailability of metals in soil was traditionally evaluated using chemical extraction methods (sodium acetate, CaCl_2_ [6], hydrochloric acid, *aqua regia*) or by measuring soil solution concentrations. However, the accuracy of the results depended on the physicochemical characteristics of the soil (*i.e.*, temperature, pH, redox conditions, organic matter, and soil clay type). Moreover, these equilibrium-based approaches do not account for depletion at the root-soil interface and depletion-induced resupply from the soil.

An *in situ* DGT technique capable of quantitative measurement has been developed, which can be used to measure labile metal species in soil [7,8], sediment [9,10], and water [11] without disturbance and pre-treatment [12]. This could eliminate the analytical errors (*i.e.*, resorption and re-dissolution) common with traditional methods. As a passive sampler, DGT makes it possible to determine *in situ* with pre-concentration and to determine time-weighted average concentrations. In the uptake process, heavy metals from pore water diffuse through the diffusion layer to the binding phase [13,14]. As a surrogate of plants, DGT measurement could lower the heavy metal concentrations in the pore water in the vicinity of the DGT unit, which leads to desorption of the heavy metal from the soil into the pore water at the same time. Based on these characteristics, DGT can measure the metal bioavailability from the complete soil system [15]. Consequently, DGT was considered as a potential monitoring tool for soil with heavy metal contamination [16,17]. Research showed that the bioavailability of Cu [18,19,20] and Zn [21] determined by DGT highly correlated with the metal concentration in plants. However, there is no universal consensus on the accuracy of DGT. Due to the difference of the soil types and experimental biota as well as the contaminated species and concentrations of the heavy metals, the performance of DGT for bioavailable fractions evaluation varies a lot [22,23,24,25]. Moreover, heavy metals always occur in complex forms in the environment. For example, Cd and Pb share similar geochemical behaviour and often co-occur as environmental contaminants in soil. However, the use of DGT in relation to compound contamination of heavy metals in soil has obvious deficiencies at present. Therefore, improving the effectiveness of the DGT technique for predicting the bioavailability of Cd in contaminated soil with added Pb requires additional study.

The aim of this study was to investigate the relationship between Cd concentrations measured by DGT and traditional chemical extraction methods in Cd contaminated soil amended with the addition of Pb. The relationship between DGT-labile Cd and accumulation in plants was investigated and compared to chemical extraction methods. Linear correlations between Cd concentration in soil and plants were used to confirm the best method for predicting the concentration of labile Cd in complex soils.

## 2. Materials and Methods

### 2.1. DGT Assembly and Preparation

The DGT instrument, supplied by DGT Research Ltd. (Lancaster, UK), consists of a plastic shell enclosing a cellulose-acetate filter (protective membrane), a diffusive gel layer, and a resin (Chelex-100). A 0.45-μm pore-size filter membrane, 0.13 mm thick and with an exposed diffusion area of 3.14 cm^2^, is placed in the outermost layer. Diffusive gels and binding gels were prepared according to the procedure given in Zhang and Davison [26]. When prepared, the diffusive membrane was hydrated in demineralized water for 24 h, during which interval the water was changed 3–4 times. Until their deployment, diffusive membranes were preserved in 0.01 mol/L NaNO_3_ and resin gels were stored in demineralized water.

### 2.2. Soil Samples and Plants

The soil was collected at a depth of 0–20 cm from a vineyard in Nanjing Jiangxinzhou. Larger impurities were taken out and the dry soil was ground and sieved through 2 mm mesh. The air-dried homogenized soil sample was then used for wheat (*Bainong AK58*) and maize (*Zhengdan 958*) cultivation. The basic physicochemical properties of soil and the heavy metal content were determined according to the procedure of Sun *et al.* [27]. The initial soil was tested for the following chemical properties: pH (6.5), organic matter (OM, 2.5%), cation exchange capacity (CEC, 26.9 cmol/kg), and total Cd, Zn, Pb, and Cu concentrations (0.22, 130.8, 19.04, and 64.4 mg/kg, respectively).

The soil samples were spiked with cadmium chloride and lead nitrate to achieve 4 mg/kg Cd with different concentrations of soil Pb (0, 20, 40, 80, 160, 320, and 640 mg/kg), while non-treated soil (CK) served as the control. Nitrogen (0.15 g/kg), phosphorous (0.1 g/kg), and potassium (0.15 g/kg) were added to the control and treated soil, which was then incubated for six weeks at room temperature. During this time, the soil moisture was maintained by adding deionized water, and fully mixed every 3–4 d to ensure equilibration between the soil and the added solution.

### 2.3. Pot Experiment

The control and treated soils were potted (0.75 kg moist soil per pot). The plump seeds were disinfected with 75% alcohol for 10 min, then wheat and maize seeds were soaked in distilled water for 1 or 3 d. Five seeds of maize and fifteen seeds of wheat were sown in pots filled with 0.75 kg of either non-treated or spiked soils. After germination, the number of plants in each pot was reduced to three seedlings for maize and ten seedlings for wheat. To ensure the accuracy of the experiment, each combination of added Cd and Pb concentration were treated in triplicate. After 35 days, each plant was separated into shoots and roots. Plants were rinsed first with tap water, and then with deionized water, in order to clean their surface. The roots were soaked in 20 mmol/L Na-EDTA solution for 15 min to remove Cd^2+^ adsorbed to the surface and then washed with distilled water. Samples were placed in an oven at 105 °C for 20 min, and then subjected to 70 °C drying, until reaching constant weight. The dry weights were recorded for the determination of total heavy metal. After harvest, the remaining soils were air-dried at room temperature and sieved with 2-mm stainless-steel mesh for the following analysis of bioavailable Cd in soils.

### 2.4. DGT Experiments

The DGT-Cd determination of metals in the soils was performed according to the procedure described by Luo *et al.* [28]. The process involved four steps:

(1) *Pretreatment of the soil sample*: each soil sample (80 g) was weighed in a 100 mL plastic container and mixed with deionized water to 40% maximum water holding capacity (MWHC); 48 h later, water was added to achieve 80% MWHC and the resulting slurries were allowed to equilibrate at ambient temperature for 24 h before DGT deployment.
(2) *DGT deployment*: the assembled DGT devices were gently placed on the soil surface of each pot for 24 h, but the gel films were not squeezed. The containers were closed, and Petri dishes with wet cellulose were placed in the containers to retain the soil moisture. Three replicates per pot were kept at 25 °C for 24 h.
(3) *DGT retrieval and elution*: after 24 h, all of the DGT devices were retrieved and rinsed with deionized water. The binding gel layers were removed from the DGT units, placed in polyethylene vials, and eluted in 1 mL of 1 mol/L HNO_3_ for 24 h. The Cd concentration in the extractant was determined by flame atomic adsorption spectrophotometry (Z-81001, Hitachi, Hitachi, Japan).
(4) *DGT calculation*: The concentrations of Cd accumulated by the DGT devices were calculated according to Equation (1):
  (1) $$C_{\text{DGT}}=\frac{M\Delta g}{DAt}$$

where *C*_DGT_ represents the metal supply from the soil solution and solid phases (mg/L); *M* is the accumulated mass of metal on the binding gel (µg); Δ*g* is the thickness of the diffusion layer(cm); and *D* is the diffusion coefficient of metal ion (cm^2^/s). The *D* value was temperature corrected. Here, *A* is the area of the resin gel exposed to the diffusion flux (cm^2^) and t is the deployment time (s).

### 2.5. Soil Solution Concentration

After completion of the DGT deployment, soil solution was obtained from slurries by centrifugation at 4000 rpm for 20 min. Subsequently supernatants were filtered using 0.45-μm cellulose membrane for measurement of the Cd concentration.

### 2.6. Single-Solvent Process

For the single-leaching procedure commonly used for measurement of Cd content in soil, 0.5 g of dried, sieved soil was shaken in a 50 mL centrifuge tube with 20 mL of 0.11 mol/L acetic acid (HOAc) at 60 rpm and ambient temperature for 16 h [29]. Then, 2.0 g of dried, sieved soil was shaken in a 50 mL centrifuge tube with 20 mL of 0.05 mol/L EDTA at 60 rpm and room temperature for 2 h [30]. Next, 2.0 g of dried, sieved soil was shaken in a 50 mL centrifuge tube with 20 mL of 0.01 mol/L CaCl_2_ at 60 rpm and ambient temperature for 2 h [31]. Finally, 4.0 g of dried, sieved soil was shaken in a 50 mL centrifuge tube with 20 mL of 1 mol/L sodium acetate (NaOAc) at 60 rpm and room temperature for 2 h [32]. The detailed operations are listed in Table 1. All the extracts were immediately filtered through a membrane filter (0.45-μm pore size) and the filtrate was refrigerated for determination of Cd.

### 2.7. Determination of Cd in Plants

The shoots and roots were crushed using a plant-grinding machine and sieved through 2 mm mesh. The samples were digested with HNO_3_/HClO_4_ then for the determination of Cd contents in plant tissues.

## 3. Results and Discussion

### 3.1. Wheat and Maize Growth Response to Cd and Pb

**Figure 1:**
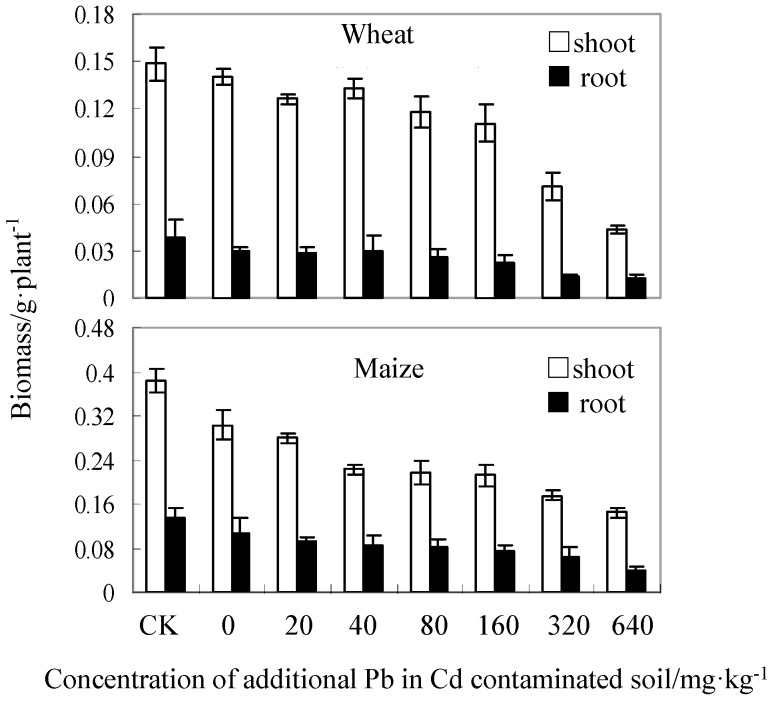
Biomass of wheat and maize grown in Cd-contaminated soil at various concentrations of added Pb. CK represents the control group.

As soil contaminants, Cd and Pb are highly mobile and toxic. Previous studies showed that when the contamination includes Cd and Pb, the combination has a negative synergistic effect on plant growth. The same conclusion was drawn in this study. Soil contaminated only with Cd (4 mg/kg) had no influence on wheat, but the shoot and root biomass of the maize was reduced by 22.15 and 23.19% respectively (Figure 1). The inhibitory effect of Cd combined with Pb on plants becomes more obvious with increasing Pb concentration, and the toxicity for maize was more significant than for wheat.

### 3.2. Cd Uptake by Plants

**Figure 2:**
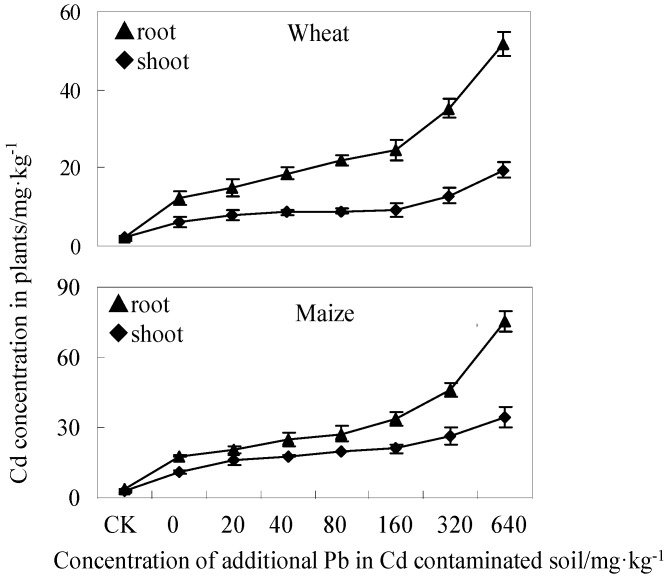
Cd concentration in the shoots and roots of wheat and maize grown in Cd-contaminated soil at various concentrations of added Pb. CK represents the control group.

The uptake and distribution of Cd in wheat and maize are presented in Figure 2. The concentration of Cd in both wheat and maize was higher in the treated group than in the control. Cd was mainly accumulated in roots, and this was previously reported for *Agrostis tenuis* [33]. The concentration of Cd in both parts of the wheat and maize plants increased with increasing soil-Pb content and surged at high Pb-concentrations. These results showed that the presence of Pb in Cd-contaminated soil promoted the Cd uptake and accumulation by wheat and maize, and even enhanced the bioavailability and ecotoxicity of Cd in soil.

### 3.3. DGT Measurement and Soil Solution Concentration

**Figure 3:**
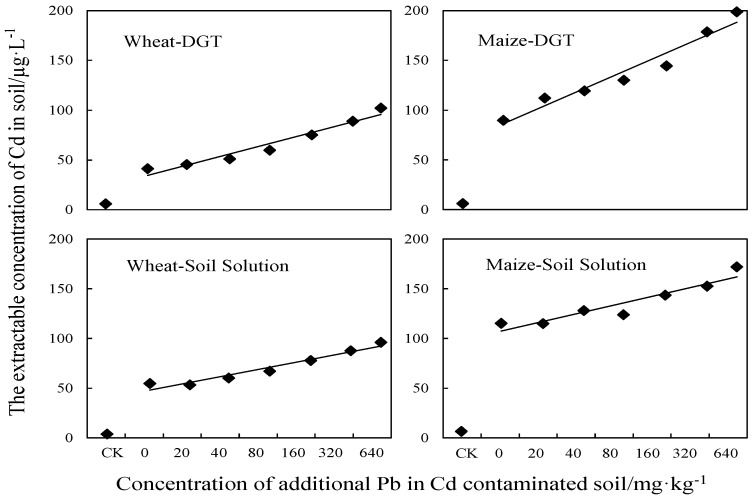
Bioavailable Cd by DGT and Soil Solution in wheat/maize-grown soils. CK represents the control group.

The resin impregnated with Chelex-100 (SIGMA, St. Louis, MS, USA) (in the DGT device) showed stronger adsorption than other complexants, for determining the bioavailability of target elements [34]. In the uptake process, heavy metals from the bioavailable reservoir diffuse through the diffusion layer to the resin gel. As in the uptake by organisms, DGT uptake also lowers the heavy metal concentrations in the pore water surrounding the DGT unit. This leads to local depletion of the bioavailable fraction, which induces resupply from the solid phase. Consequently, DGT can dynamically measure the heavy metal bioavailability from the complete soil system immediately surrounding the device. The soil solution could directly reflect the status of bioavailable fractions, so toxicity or bioaccumulation of heavy metals is usually evaluated using the soil solution concentration [35]. As shown in Figure 3, the DGT-labile Cd concentration (*C*_DGT_) and the concentration of Cd in soil solution (*C*_sol_), nearly linearly increased with increasing concentration of the added Pb. For wheat *R*^2^ = 0.9578 and 0.8839 was determined by DGT and soil solution respectively. For maize *R*^2^ = 0.9235 and 0.8801 was measured by DGT and soil solution. As the result of dynamic measurement by DGT and the static balance reflected in the soil solution, it was shown that the increased Pb concentration could significantly increase the content of extractable Cd in soil. This result is in agreement with Lin *et al.* [36], who found that Pb treatment affected the chemical form of metals in soil and increased the exchangeable-Cd content. The DGT-measured Cd as well as labile Cd in the soil solution of the maize-grown soil was obviously higher than that of the wheat-grown soil. Black [16] have also revealed the effects of different plant species on the labile fractions distribution. The differences of the rhizosphere environment and the rhizospheric microorganisms might contribute to the redistribution of the labile fractions, which resulted in the significantly different values obtained by the indicators.

### 3.4. Extractable-Cd Determined by Single Extraction Methods

**Figure 4:**
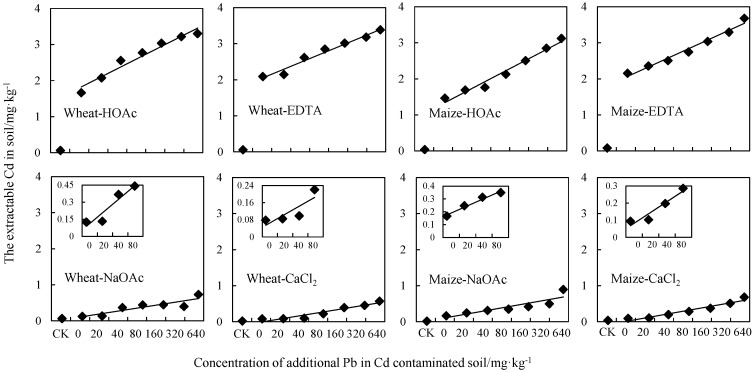
Bioavailable Cd measured in wheat and maize soil using four extraction methods. CK represents the control group.

The bioavailable concentrations of Cd in soil extracted by four different extractants (EDTA, HOAc, CaCl_2_, and NaOAc) are shown in Figure 4. Similar to the results of DGT and *C*_sol_, the extractable concentration of Cd in soil by all extractants increased nearly linearly with increasing concentration of added Pb. We found significant correlation (for maize *R*^2^ = 0.7591, 0.8960, 0.9529, and 0.8819; for wheat *R*^2^ = 0.7399, 0.8271, 0.9187, and 0.8713) between extractable-Cd measured by the four different methods and the concentration of Pb in soil. This confirmed that the addition of Pb in the soil can increase the bioavailability of Cd beyond that of static equilibrium. For extraction by the four different reagents, the concentration of Cd extracted decreased in the order EDTA > HOAc > NaOAc > CaCl_2_ and this corresponded with the results reported by other researchers [37]. The concentration of extracted-Cd mainly depended on the properties of the extractants chosen. As a chelating agent, EDTA extracted exchangeable, mineral-combined, and organically bound Cd [38]. The HOAc, as a weak acid extractant, obtained Cd fractions which included soluble and exchangeable Cd, as well as Cd associated with calcium carbonate and minerals. Because NaOAc and CaCl_2_ are both the neutral salt extractants, they mainly extracted easily exchangeable Cd on the soil-mineral surfaces with exchangeable cations [38]. However, the higher concentration of Cd extracted by NaOAc differed from the results of Novozamsky [31].

All the indicators showed the increasing bioavailability of Cd with the increasing mass of Pb addition. As we know, the Cd fractions have relatively higher mobility compared to the Pb fractions [24,39]. Moreover, Pb as one of the most common environmental contaminants, has limited solubility in soil and availability for biota accumulation because of the complexation with organic matter and sorption on the oxides and clays [40]. Consequently, the additional Pb could led to the decrease of the soil capacity for Cd adsorption. The results show the increasing concentration of Pb addition could significantly increase the Cd fractions released from the solid phase to the solution.

### 3.5. Comparison of DGT with Chemical Extraction for Cd Bioavailability

Linear correlation between labile-metal concentrations in soil and the metal content in plants has been widely used for evaluating soil-testing methods. As shown in Table 2, significant positive relationships (*p* < 0.01; *p* < 0.05) between Cd concentration in soil measured by six methods and Cd content in shoots and roots of wheat and maize were found in the experiment. Highly positive correlation coefficients were obtained by all methods and there was no obvious difference between DGT and other methods. This is the results of the high mobilization of labile Cd fractions in soil solution as well as the fast resupply from solid phase to soil solution [41]. Cd is characterized by a short response time for resupply from the solid phase and a low solid-liquid distribution coefficient. All these features of Cd determined the ease of its extractability by all chosen methods.

*C*_DGT_ not only includes ions dissolved in soil solution, but also metals released from soil solid phase. This resulted in the better performance of DGT for labile Cd evaluation compared to that of traditional chemical reagents. Using a multi-factor analysis method, Tian *et al.* [42] and Song *et al.* [20] showed that the essential physicochemical properties of soil (e.g., organic content, soil texture) did not affect the DGT technology. However, traditional chemical extraction methods are easily influenced by them, so DGT technology is a better method for predicting heavy metal bioavailability and uptake by plants. Zhang *et al.* [15] reported that fluxes of copper measured by DGT related well to copper uptake by *Lepidium heterophyllum*. Dočekalová *et al.* [43] found high correlation of flux to radish plant with flux to DGT for Cd (*R*^2^ = 0.994). Nolan *et al.* [25] found a high coefficient of determination (*R*^2^ = 0.90) between DGT uptake and Cd contents in wheat. Bade *et al.* [44] demonstrated that DGT uptake was linearly correlated with the total heavy metal concentrations in soil, and that DGT can be used as a biomimetic surrogate for predicting bioavailable concentrations of heavy metals in soil.

## 4. Conclusions

The combination of Cd and Pb had a negative synergistic effect on the growth of plants. The addition of Pb can significantly enhance Cd accumulation in the shoots and roots of maize and wheat. Cd tolerance varies by plant species, and the results showed that maize was more sensitive to increasing concentration of heavy metals (Cd and Pb) than wheat, although the growth of both species was inhibited. The concentrations of different indicators measured Cd in soil increased with increasing addition of Pb. Pearson coefficients showed that the Cd concentrations in soil positively correlated with the Cd accumulation in plants. Moreover, the values determined by DGT, *C*_sol_, and CaCl_2_ correlated better than the other three methods. It appears that the DGT technique could be used as a physical surrogate for plant uptake, thus offering the possibility of a simple test procedure for measurement of bioavailable heavy metals in soil.

## Figures and Tables

**Table 1:** Determination of Cd in plants.

Extractant	Procedure	References
EDTA	2.0 g of soil was extracted with 20 mL of 0.05 mol·L^−1^ EDTA adjusted using an ammonia solution to pH = 7.0 and shaken for 2 h	Feng *et al.* [30]
HOAc	0.5 g of soil was extracted with 20 mL of 0.11 mol·L^−1^ HOAc and shaken for 16 h (overnight)	Quevauviller [29]
NaOAc	4.0 g of soil was extracted with 20 mL of 1 mol·L^−1^ NaOAc and shaken for 2 h	Kaplan *et al.* [32]
CaCl_2_	2.0 g of soil was extracted with 20 mL of 0.01 mol·L^−1^ CaCl_2_ and shaken for 3 h	Novozamsky *et al.* [31]

**Table 2:** Linear correlation coefficients (*r*) between Cd concentrations in the plant tissues and bioavailable concentrations of Cd measured by different methods in soils.

Plant Species	Plant Tissues	*C*_DGT_	*C*_sol_	HOAc	EDTA	NaOAc	CaCl_2_
Wheat	Shoot	0.944 **	0.923 **	0.850 **	0.841 **	0.882 **	0.960 **
	Root	0.931 **	0.905 **	0.789 **	0.763 **	0.857 **	0.943 **
Maize	Shoot	0.994 **	0.971 **	0.899 **	0.900 **	0.891 **	0.925 **
	Root	0.915 **	0.968 **	0.874 **	0.829 **	0.801 **	0.901 **

Note: ** Correlation is significant at the 0.01 level.

## References

[1] McLaughlin M.J., Parker D.R., Clarke J.M. (1999). Metals and micronutrients—Food safety issues. Field Crop. Res..

[2] International Agency for Research on Cancer (1993). Beryllium, Cadmium, Mercury, and Exposures in the Glass Manufacturing Industry.

[3] Wuana R.A., Okieimen F.E. (2011). Heavy metals in contaminated soils: A review of sources, chemistry, risks and best available strategies for remediation. ISRN Ecol..

[4] Sánchez-Martín M.J., García-Delgado M., Lorenzo L.F., Rodríguez-Cruz M.S., Arienzo M. (2007). Heavy metals in sewage sludge amended soils determined by sequential extractions as a function of incubation time of soils. Geoderma.

[5] Ytreberg E., Karlsson J., Hoppe S., Eklund B., Ndungu K. (2011). Effect of organic complexation on copper accumulation and toxicity to the estuarine Red Macroalga *Ceramium tenuicorne*: A test of the free ion activity model. Environ. Sci. Technol..

[6] Houba V.J.G., Lexmond T.M., Novozamsky I., van der Lee J.J. (1996). State of the art and future developments in soil analysis for bioavailability assessment. Sci. Total Environ..

[7] Davlson W., Zhang H. (1994). *In situ* speciation measurements of trace components in natural waters using thin-film gels. Nature.

[8] Jansen B., Nierop K.G., Verstraten J.M. (2002). Influence of pH and metal/carbon ratios on soluble organic complexation of Fe(II), Fe(III) and Al(III) in soil solutions determined by diffusive gradients in thin films. Anal. Chim. Acta.

[9] Davison W., Fones G.R., Grime G.W. (1997). Dissolved metals in surface sediment and a microbial mat at 100-μm resolution. Nature.

[10] DeVries C.R., Wang F. (2003). *In situ* two-dimensional high-resolution profiling of sulfide in sediment interstitial waters. Environ. Sci. Technol..

[11] Omanović D., Pižeta I., Vukosav P., Kovács E., Frančišković-Bilinski S., Tamás J. (2015). Assessing element distribution and speciation in a stream at abandoned Pb–Zn mining site by combining classical, *in-situ* DGT and modelling approaches. Sci. Total Environ..

[12] Davison W., Zhang H. (2012). Progress in understanding the use of diffusive gradients in thin films (DGT)—Back to basics. Environ. Chem..

[13] Davison W., Zhang H., Miller S. (1994). Developing and applying new techniques for measuring steep chemical gradients of trace metals and inorganic ions at the sediment-water interface. Mineral. Mag. A.

[14] Zhang H., Davison W., Miller S., Tych W. (1995). *In situ* high resolution measurements of fluxes of Ni, Cu, Fe, and Mn and concentrations of Zn and Cd in porewaters by DGT. Geochim. Cosmochim. Acta.

[15] Zhang H., Zhao F.J., Sun B., Davison W., Mcgrath S.P. (2001). A new method to measure effective soil solution concentration predicts copper availability to plants. Environ. Sci. Technol..

[16] Black A., McLaren R.G., Reichman S.M., Speir T.W., Condron L.M. (2011). Evaluation of soil metal bioavailability estimates using two plant species (*L. perenne* and *T. aestivum*) grown in a range of agricultural soils treated with biosolids and metal salts. Environ. Pollut..

[17] Pérez A.L., Anderson K.A. (2009). DGT estimates cadmium accumulation in wheat and potato from phosphate fertilizer applications. Sci. Total Environ..

[18] Tandy S., Mundus S., Yngvesson J., de Bang T.C., Lombi E., Schjørring J.K., Husted S. (2011). The use of DGT for prediction of plant available copper, zinc and phosphorus in agricultural soils. Plant Soil.

[19] Qiu H., Gu H.-H., He E.-R., Wang S.-H., Qiu R.-L. (2012). Attenuation of metal bioavailability in acidic multi-metal contaminated soil treated with fly ash and steel slag. Pedosphere.

[20] Song J., Zhao F.J., Luo Y.M., McGrath S.P., Zhang H. (2004). Copper uptake by *Elsholtzia splendens* and *Silene vulgaris* and assessment of copper phytoavailability in contaminated soils. Environ. Pollut..

[21] Zhang H., Lombi E., Smolders E., McGrath S. (2004). Kinetics of Zn release in soils and prediction of Zn concentration in plants using diffusive gradients in thin films. Environ. Sci. Technol..

[22] Soriano-Disla J.M., Speir T.W., Gómez I., Clucas L.M., McLaren R.G., Navarro-Pedreño J. (2010). Evaluation of different extraction methods for the assessment of heavy metal bioavailability in various soils. Water Air Soil Pollut..

[23] Cornu J.Y., Denaix L. (2006). Prediction of zinc and cadmium phytoavailability within a contaminated agricultural site using DGT. Environ. Chem..

[24] Almås Å.R., Lombnæs P., Sogn T.A., Mulder J. (2006). Speciation of Cd and Zn in contaminated soils assessed by DGT-DIFS, and WHAM/Model VI in relation to uptake by spinach and ryegrass. Chemosphere.

[25] Nolan A.L., Zhang H., McLaughlin M.J. (2005). Prediction of zinc, cadmium, lead, and copper availability to wheat in contaminated soils using chemical speciation, diffusive gradients in thin films, extraction, and isotopic dilution techniques. J. Environ. Qual..

[26] Zhang H., Davison W. (1995). Performance characteristics of diffusion gradients in thin films for the *in situ* measurement of trace metals in aqueous solution. Anal. Chem..

[27] Sun Q., Zhang L., Ding S., Li C., Yang J., Chen J., Wang P. (2015). Evaluation of the diffusive gradients in thin films technique using a mixed binding gel for measuring iron, phosphorus and arsenic in the environment. Environ. Sci. Processes Impacts.

[28] Luo J., Zhang H., Zhao F.J., Davison W. (2010). Distinguishing diffusional and plant control of Cd and Ni uptake by hyperaccumulator and nonhyperaccumulator plants. Environ. Sci. Technol..

[29] Quevauviller P. (1998). Operationally defined extraction procedures for soil and sediment analysis I. Standardization. TrAC Trends Anal. Chem..

[30] Feng M.H., Shan X.Q., Zhang S.Z., Wen B. (2005). Comparison of a rhizosphere-based method with other one-step extraction methods for assessing the bioavailability of soil metals to wheat. Chemosphere.

[31] Novozamsky I., Lexmond T.M., Houba V.J.G. (1993). A single extraction procedure of soil for evaluation of uptake of some heavy metals by plants. Int. J. Environ. Anal. Chem..

[32] Kaplan O., Yaman M., Kaya G. (2009). Distribution of nickel in different phases of soil samples and plant parts taken from serpentine and copper mining area. Asian J. Chem..

[33] Dahmani-Muller H., Van Oort F., Gelie B., Balabane M. (2000). Strategies of heavy metal uptake by three plant species growing near a metal smelter. Environ. Pollut..

[34] Harper M.P., Davison W., Zhang H., Tych W. (1998). Kinetics of metal exchange between solids and solutions in sediments and soils interpreted from DGT measured fluxes. Geochim. Cosmochim. Acta.

[35] Forsberg L.S., Kleja D.B., Greger M., Ledin S. (2009). Effects of sewage sludge on solution chemistry and plant uptake of Cu in sulphide mine tailings at different weathering stages. Appl. Geochem..

[36] Lin Q., Chen Y.X., Chen H.M., Yu Y.L., Luo Y.M., Wong M.H. (2003). Chemical behavior of Cd in rice rhizosphere. Chemosphere.

[37] Paya-Perez A., Sala J., Mousty F. (1993). Comparison of ICP-AES and ICP-MS for the analysis of trace elements in soil extracts. Int. J. Environ. Anal. Chem..

[38] McLaughlin M.J., Zarcinas B.A., Stevens D.P., Cook N. (2000). Soil testing for heavy metals. Commun. Soil Sci. Plant Anal..

[39] Kim K.R., Owens G., Naidu R. (2009). Heavy metal distribution, bioaccessibility and phytoavailability in long-term contaminated soils from Lake Macquarie, Australia. Aust. J. Soil Res..

[40] McBride M.B. (1994). Environmental Chemistry of Soils.

[41] Manouchehri N., Besançon S., Bermond A. (2011). Kinetic characterizing of soil trace metal availability using Soil/EDTA/Chelex mixture. Chemosphere.

[42] Tian Y., Wang X., Luo J., Yu H., Zhang H. (2008). Evaluation of holistic approaches to predicting the concentrations of metals in field-cultivated rice. Environ. Sci. Technol..

[43] Dočekalová H., Škarpa P., Dočekal B. (2015). Diffusive gradient in thin films technique for assessment of cadmium and copper bioaccessibility to radish (*Raphanus sativus*). Talanta.

[44] Bade R., Oh S., Shin W.S. (2012). Diffusive gradients in thin films (DGT) for the prediction of bioavailability of heavy metals in contaminated soils to earthworm (*Eisenia foetida*) and oral bioavailable concentrations. Sci. Total Environ..

